# Severe Inflammatory Heart Disease in Children With Lupus: A Case Series

**DOI:** 10.1016/j.cjcpc.2024.05.005

**Published:** 2024-05-31

**Authors:** Geena Kim, Deborah M. Levy, Dawn Nicolson, Sunghoon Minn, Andrea Knight, Linda T. Hiraki, Aine Lynch, Emilie Jean-St-Michel, Jenna Ashkanase, Aamir Jeewa

**Affiliations:** a Department of Pediatrics, Chungnam National University Sejong Hospital, Chungnam National University School of Medicine, Sejong, Korea; b Division of Cardiology, Labatt Family Heart Centre, Hospital for Sick Children, Toronto, Ontario, Canada; c Division of Rheumatology, Hospital for Sick Children, Toronto, Ontario, Canada; d Division of Cardiology, Department of Pediatrics, Morgan Stanley Children’s Hospital of New York Presbyterian, Columbia University Medical Center, New York, New York, USA; e Department of Pediatrics, University of British Columbia, Vancouver, British Columbia, Canada; f Division of Cardiology, McMaster Children's Hospital, Hamilton, Ontario, Canada

**The cardiac manifestations of systemic lupus erythematosus (SLE) can confer significant morbidity. Severe myocarditis is a rare, but important, cause of mortality. In this report, we describe 3 cases of adolescents with severe cardiac manifestations of SLE myocarditis. This case series demonstrates the varying and potentially severe manifestations of SLE myocarditis. Early recognition and treatment could potentially improve the outcomes of patients with SLE myocarditis.**

Myocarditis is an inflammatory disease of the heart that can occur due to both infectious and noninfectious causes. Noninfectious causes of myocarditis include autoimmune inflammatory disease, hypersensitivity, medications, and toxins.^1^ Systemic lupus erythematosus (SLE) causes severe systemic inflammation that can include the cardiovascular system, most commonly manifesting as pericarditis. Although rare, myocarditis has been reported as a manifestation of SLE.^2^^,^^3^ The clinical presentation of myocarditis can range from mild chest pain to cardiogenic shock requiring mechanical circulatory support, and thus, early diagnosis is important in patients with SLE to facilitate timely treatment.

Herein, we report a case series of SLE myocarditis in adolescents at our hospital, including their clinical characteristics, course, treatment, and outcomes.

## Case 1

**Figure 1:**
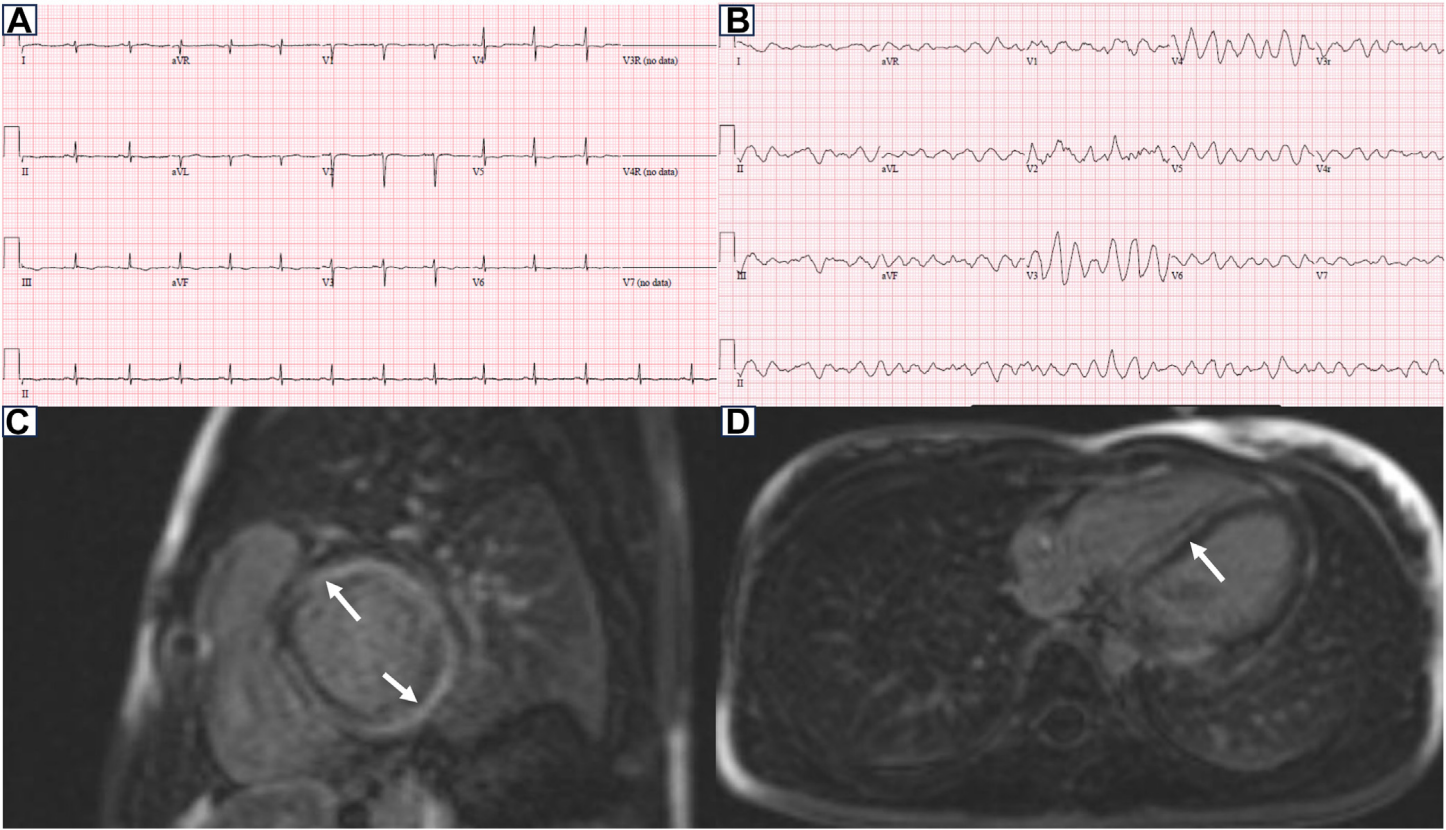
Electrocardiogram for the patient described in case 1 and cardiac magnetic resonance imaging of the patient described in case 2. Low-voltage QRS complexes and diffuse T-wave flattening (**A**) and ventricular fibrillation (**B**), sagittal (**C**), and axial view (**D**) revealing myocardial oedema with dense late gadolinium enhancement (**arrow**).

A 16-year-old girl was transferred from a community hospital after presentation with 2 days of chest pain. She had received a COVID-19 vaccination 1 day prior, and, importantly, her chest pain symptoms had started before the administration of the vaccine. Her medical history was remarkable for a diagnosis of vitiligo within the previous year. Family history was noncontributory. In the emergency room (ER), her vital signs were stable. An electrocardiogram showed low-voltage QRS complexes and diffuse T-wave flattening (Fig. 1A). Echocardiography performed at the bedside showed moderate-to-severely reduced left ventricular systolic function, with an ejection fraction of 30%, and mild mitral regurgitation. Her laboratory investigations revealed an elevated troponin I (14,523 ng/L; normal <30.9 ng/L) and N-terminal pro b-type natriuretic peptide (4109.2 ng/L; normal <125.0 ng/L). In the ER, she suffered a cardiac arrest secondary to ventricular fibrillation (VF) (Fig. 1B). Emergency extracorporeal cardiopulmonary resuscitation and subsequent balloon atrial septostomy for left heart decompression were performed. Methylprednisolone pulse therapy was administered for 5 days in addition to 1 dose of intravenous immunoglobulin (IVIg) and lidocaine for ventricular arrhythmia. No infectious or metabolic causes of myocarditis were identified. Additional workup showed low C3 and C4 (0.47 g/L; normal 0.83-1.52 g/L and 0.08 g/L; normal 0.13-0.37 g/L, respectively) and a positive antinuclear antibody (1:640), with a speckled pattern. Further serologic testing on samples drawn before IVIg demonstrated high titre positive anti-Smith, anti-ribonucleoprotein, anti-Ro60, and anti-La, suggesting an autoimmune inflammatory etiology (Table 1). Further history revealed a discoid rash 4 years prior with a subsequent diagnosis of SLE. Hydroxychloroquine had been initiated but was discontinued by the family, and she had been lost to follow-up.

**Table 1:** Patient characteristics

Variables	Patient 1	Patient 2	Patient 3
Sex	Female	Female	Female
Age (y)	16	14	17
Cardiac symptoms	Chest pain, syncope, VF arrest	Shortness of breath, VF arrest	Respiratory distress
Lupus symptoms	Discoid lupus	Malar rash, alopecia	Rash, polyarthritis, renal disease, CNS disease, cytopaenia
Diagnosis time from onset of lupus	4 y	Concomitant	6 y
Laboratory			
C3, C4	Low	Normal	Low
ANA	Positive	Positive	Positive
Ro	Positive	Negative	Negative
La	Positive	Negative	Negative
RNP	Positive	Negative	Positive
Smith	Positive	Negative	Positive
Antiphospholipid antibodies	Negative	Negative	Negative
Anti-dsDNA	Negative	Positive	Positive
Renal involvement	None	None	Lupus nephritis IV and V
Echocardiography	EF 30%	EF 23%	EF 23%, large PE
Cardiac MRI	Diffuse myocarditis	Mid wall, basal myocarditis	Not done
Cardiac support for procedure	ECMO	None	Pericardiocentesis
Treatment	IVIg, steroid, MMF, hydroxychloroquine	Steroid, metoprolol, hydroxychloroquine	Steroid, MMF
Outcome	Full recovery	Full recovery	Deceased
Other complications of SLE	None	None	Encephalopathy, ESKD

ANA, antinuclear antibody; Anti-dsDNA, Anti–double stranded DNA; CNS, central nervous system; ECMO, extracorporeal membrane oxygenation; EF, ejection fraction; ESKD, end stage kidney disease; IVIg, intravenous immunoglobulin; MMF, mycophenolate mofetil; MRI, magnetic resonance imaging; PE, pericardial effusion; RNP, ribonucleoprotein; SLE, systemic lupus erythematosus; VF, ventricular fibrillation.

She was weaned from extracorporeal membrane oxygenation after 8 days. Ventricular function improved with full recovery 1 month after admission. She required 20 days of haemodialysis for kidney injury with a gradual wean thereafter. Corticosteroids were tapered over 6 months, and mycophenolate mofetil (MMF) was initiated (1200 mg/m^2^/d), in addition to hydroxychloroquine (5 mg/kg/d). At the most recent follow-up, 2 years after the cardiac event, she had clinically and serologically quiescent SLE with normal cardiac function.

## Case 2

A 14-year-old girl was transferred to our hospital after resuscitation from a cardiac arrest secondary to VF while at summer camp. A week before presentation, she had 1 episode of emesis and experienced shortness of breath 2 days before the cardiac arrest. She was previously healthy with an unremarkable family history. After resuscitation, the initial echocardiogram demonstrated severely reduced left ventricular systolic function. She also developed polymorphic premature ventricular contractions that were treated with lidocaine. Initial troponin I (1537 ng/L; normal <30.9 ng/L) and high-sensitivity C-reactive protein were elevated (19.2 mg/L; normal <1.7 mg/L). Investigations for an infectious etiology were negative, and she had an isolated elevated antinuclear antibody level (1:640). After admission to the intensive care unit, she was haemodynamically stable without the requirement for inotropic support and was extubated within 3 days. Her cardiac magnetic resonance imaging revealed severe mid-wall myocardial oedema of the basal two-thirds of the left ventricle, with the central area of the oedematous myocardium showing dense late gadolinium enhancement, consistent with myocarditis (Fig. 1C and D). She was treated with methylprednisolone (2 mg/kg/d) for 5 days and a β-blocker for her myocarditis and arrhythmia, with the course of steroids weaned over 3 months. After discharge, she underwent further work-up for SLE and was noted to have a malar rash, a history of alopecia, an elevated anti–double stranded DNA (74.8 IU/mL; normal <27 IU/mL), and borderline anticardiolipin antibody (21.2 IU/mL). She was commenced on hydroxychloroquine therapy and has remained stable without further active SLE or cardiac progression 3 years after the cardiac event.

## Case 3

An 11-year-old girl was diagnosed with SLE when she presented with arthritis, lymphopaenia, haemolytic anaemia, and autoantibodies. After her family’s relocation out of the country, she developed lupus nephritis. On representation to our centre, she had recalcitrant hypertension with significant renal insufficiency. She was treated with immunosuppressive therapy, including steroids, MMF, and cyclophosphamide. Eventually, she progressed to end-stage renal disease requiring haemodialysis, which was accompanied by severe hypertension requiring multiple antihypertensive medications.

Cardiac evaluation revealed intermittent borderline left ventricular dysfunction and moderate-to-large pericardial effusions that reaccumulated after 2 pericardiocentesis procedures.

At 16 years of age, she presented to the ER with respiratory distress. Echocardiography showed severely decreased left ventricular function with an ejection fraction of 26% and a large pericardial effusion. Laboratory tests demonstrated elevated troponin I (940.1 ng/L; normal <30.9 ng/L), a high-sensitivity C-reactive protein (5.7 mg/L; normal <1.7 mg/L), hypocomplementaemia, elevated anti–double stranded DNA antibody, and elevated IgG levels. She was started on inotropes and IV methylprednisolone for 3 days, along with daily haemodialysis. After treatment, follow-up echocardiography showed normalized systolic function with a moderate pericardial effusion, and she was discharged from the hospital on MMF and tapered prednisone.

On serial echocardiography, ventricular function fluctuated from mild to severely decreased, depending on multiple factors, such as refractory hypertension, end-stage kidney disease, and SLE activity, for approximately 9 months. Despite relative control of her renal disease and hypertension via dialysis and medical therapy, she would continue to have episodic development of pericardial effusions and depressed ventricular function, suggestive of an ongoing myocardial response to the inflammatory state. At her last admission at 17 years, she presented to the ER with shortness of breath, and left ventricular systolic function was found to be severely reduced with a moderate-to-large pericardial effusion and cardiac tamponade physiology. She received steroids and dobutamine followed by milrinone but then developed seizures. Palliative management was undertaken, and she died shortly thereafter.

## Discussion

Cardiac involvement is one of the most serious clinical manifestations of SLE. Herein, we present 3 cases of SLE-associated myocarditis that emphasize the variability in presentation, timing, and outcomes, as well as the significant associated mortality. Myocarditis is hypothesized to be an immune complex-mediated vascular phenomenon that does not lead to direct involvement of myofibrils but instead to complement activation and inflammation; it was suggested that myocarditis and nephritis partially overlap based on similar mechanisms.^4^ There is a higher incidence of SLE myocarditis reported in children than adults.^5^ In one large cohort series of children with SLE, myocarditis occurred in approximately 1% compared with 0.3% in the adult group;^5^ thus paediatric cardiac care teams should retain a high index of clinical suspicion for SLE in patients presenting with acute myocarditis.

Our case series of SLE-associated myocarditis in adolescents includes patients with diverse systemic manifestations of SLE, but with the consistent theme of severe cardiac presentations requiring advanced cardiac support. Despite limited data, existing publications are indicative of poor outcomes in SLE myocarditis, with mortality varying from 4% to 20%. However, with appropriate intervention, up to 60% make a full recovery and relapses on therapy are rare (4%).^6^ It is thus critical to confirm the diagnosis of SLE expeditiously as immunomodulatory therapies may prevent further cardiac decompensation and escalation of support.

Two of the 3 adolescents in our series presented with VF-related cardiac arrest, similar to 1 prior reported case.^2^ Though uncommon, consideration for increased screening with regards to arrhythmia burden or nonsustained ventricular tachycardia may be warranted in children with SLE, especially in those with prior myocarditis episodes. In addition, the inflammatory response after the COVID-19 vaccination has been well documented to date. The first case presented in this series had been administered the vaccine before presentation to hospital; however, the chest pain symptoms, associated with her myocarditis, preceded that event, thus making it unlikely to be related to the vaccination.

Interestingly, 2 of the patients in our series presented with cardiac manifestations much later than their initial SLE diagnosis. This has also been reported in a case by Suri et al.^7^ of an 18-year-old male patient who presented with myocarditis 6 years after SLE diagnosis. This report emphasized the use of IVIg, but not other medications, in the treatment of life-threatening complications of SLE. There are no standard medications for the management of patients with SLE myocarditis as its rarity precludes systematic trials. Thus, treatment with high-dose glucocorticoids and immunosuppressants including cyclophosphamide, rituximab, and IVIg has shown varying results. MMF and azathioprine, and perhaps belimumab, are alternative choices as they are effective for other organ manifestations of SLE. Of note, our institution ascribes to the use of pulse methylprednisolone in severe/life-threatening autoimmune diseases, even before confirmation of SLE diagnosis, and once systemic infection has been ruled out. Pulse steroids are also used in instances of severe myocarditis, especially in the setting of an arrhythmia.

The third patient in this series demonstrates the varying presentation of the inflammatory heart disease that can manifest with episodic changes in ventricular function as well as the interplay between multiorgan involvement, such as persistent anaemia and end-stage kidney disease related to lupus nephritis. One report described an 11-year-old girl with pulmonary hypertension, myocarditis, and massive pericardial effusion.^3^ In this case, the authors reported that differentiating between infectious pericarditis and SLE myocarditis based on pericardial effusion was difficult. Although our case was not accompanied by an obvious infectious source, it should be noted that SLE myocarditis could also result in pericardial effusion, regardless of an infectious origin. Moreover, SLE should be considered in the differential diagnosis of patients presenting with myocarditis and a massive pericardial effusion.

In conclusion, this case series of adolescents with severe myocarditis secondary to SLE demonstrates the potential severity of disease and need for advanced cardiac support. Moreover, our report highlights that severe myocarditis may be a presentation manifestation of SLE in children and adolescents or can manifest later as during an SLE disease flare. Prompt initiation and/or modification of immunomodulatory therapies in these patients will hopefully assist in reducing cardiac disease severity and the risk of mortality.

**Novel Teaching Points**

• In children presenting with myocarditis, it is important to rule out autoimmune etiologies as it may represent the first presentation of a systemic lupus erythematosus (SLE).
• Prompt diagnosis will result in alteration of medical management to treat the underlying autoimmune condition.
• SLE-related inflammatory heart disease may present with cardiogenic shock and/or severe haemodynamically compromised cardiac function requiring advanced heart failure support.
